# Correlation of Ultraviolet Radiation Levels With the Incidence of Cutaneous Squamous Cell and Merkel Cell Carcinomas in Non-sunbelt Locales in the United States: 2010-2017

**DOI:** 10.7759/cureus.40099

**Published:** 2023-06-07

**Authors:** Paayal S Vora, Shufeng Li, Gabriel Oh, Taylor Webb, Warren M Perry, Caroline Park, Anne Lynn S. Chang

**Affiliations:** 1 Dermatology, Northeast Ohio Medical University, Rootstown, USA; 2 Dermatology, Stanford University School of Medicine, Redwood City, USA; 3 Emergency Medicine, Emory University School of Medicine, Atlanta, USA; 4 Geriatrics, Veterans Administration Palo Alto Health Care, Palo Alto, USA

**Keywords:** rate, incidence, latitude, merkel cell carcinoma, cutaneous squamous cell carcinoma, skin cancer, ultraviolet radiation, ultraviolet index

## Abstract

Background: Non-melanoma skin cancer (NMSC) is the most common human malignancy worldwide, with increasing incidence in the United States (US). Recent environmental data have shown that ultraviolet radiation (UVR) levels have increased in the US, particularly in the higher latitudes, but the potential impact of this on NMSC incidence is not well known, despite estimates that 90% of NMSC is due to sun exposure. Our exploratory study synthesizes environmental data with demographic and clinical data to determine whether UV indices (UVIs) and non-sunbelt (non-SB) locale (latitudes >40 degrees, which comprises most of the US) might contribute to incidence rates of two types of NMSC: cutaneous squamous cell and Merkel cell carcinomas.

Methods: UVIs from 2010 to 2017 were obtained from the National Oceanic and Atmospheric Administration database and meshed with corresponding locales in the National Cancer Institute's Surveillance, Epidemiology, and End Results (SEER) database (version 8.4.0.1). Four SB and five NSB locales contained sufficient data for analysis. Linear mixed modeling was performed with the outcome variable of the age-adjusted incidence of NMSC cancer (comprised of cutaneous squamous cell carcinoma of the head and neck (CSCCHN) and Merkel cell carcinoma (MCC)), the two most common types of NMSC contained within SEER). Non-SB locale and percent of days with UVI >3 were independent variables.

Results: Percent of days with UVI >3 increased during this period, as did the overall NMSC (combined CSCCHN and MCC) skin cancer incidence, though MCC incidence alone did not increase during our study period. Environmental factors that significantly contributed to the age-adjusted overall NMSC (combined CSCCHN and MCC) cancer incidence (per 100,000 individuals) included NSB locale (b=1.227, p=0.0019) and percent of days with UVIs >3 (b=0.028, p<0.0001), as well as clinical factors of percent white race and percent male, by linear mixed modeling.

Conclusions: Our results are limited by the completeness of the NOAA and SEER databases, and do not include basal cell carcinoma. Nevertheless, our data demonstrate that environmental factors, such as latitude in NSB locale and UVI indices, can affect the age-adjusted overall NMSC (defined as CSCCHN and MCC in this study) incidence even in this relatively short period of time. Prospective studies over longer time periods are needed to identify the extent to which these findings are clinically significant so that increased educational efforts to promote sun-safe behaviors can be maximally effective.

## Introduction

Non-melanoma skin cancer (NMSC) is the most common human malignancy worldwide, with over 5.4 million estimated cases treated in the United States (US) in 2012 alone, the most recent year with comprehensive and updated statistics [1]. While NMSC includes primarily three types of cancer, namely, basal cell carcinomas (BCCs), cutaneous squamous cell carcinomas (CSCCs), and Merkel cell carcinomas (MCCs), BCCs are not routinely reported in national databases. Hence, our present study focuses on CSCC (particularly of the head and neck where most CSCCs occur [2]) and MCC.

NMSC incidence rates, including those of CSCC and MCC, have been increasing in the US over the past decades. Between 1994 and 2017, the standardized rate for the first CSCC in the US more than tripled, from 36.2 per 100,000 person-years in the time period from 1989 to 1993 to 117.8 per 100,000 person-years in 2017 [3]. In the US, the incidence rate for MCC has increased between 2000 and 2013 by 95% and from 0.5 per 100,000 person-years to 0.7 per 100,000 person-years [4]. Both CSCC and MCC incidence rates are projected to increase in the future as the proportion of older individuals in the US population increases [4,5].

While prior NMSC clinical research has focused on demographic factors, such as age, gender, and race, NMSC has long been hypothesized to be due to excessive ultraviolet radiation (UVR) in 90% of cases [6]. Cumulative UVR exposure increases with age and can be hard to accurately quantify over a lifetime. Another critical component that has been missing in much of the dermatological research literature to date has been the fact that UVR levels appear to be increasing with time.

Environmental data from 1979 to 2009 have reported that UVR (305 nm) levels reaching the US have increased, with higher latitudes demonstrating larger increases [6]. Subsequently, measures such as ultraviolet index (UVI) have become publicly available, which incorporates wavelengths from 290 to 400 nm and accounts for elevation and clouds at different times of the day (see epa.gov/sunsafety/calculating-uv-index-0). For the time period (2010-2017) and locales of this study, UVIs appear to have increased as well (though higher latitudes demonstrated lower increases). Historically, clinical research has focused on the lower latitudes, or “sunbelt” (SB) locales, despite most of the US locales being located in non-sunbelt (non-SB) locales (NSB) (latitude >40 degrees). To date, whether these increases in UVR could impact NMSC incidence is not reported.

The purpose of this study was an exploratory assessment of whether increases in UVR (as approximated by UVIs) in a variety of locales in the US could contribute to NMSC incidence, specifically CSCCHN and MCC incidences.

## Materials and methods

The percentage of days per year with UVI >3 at high noon (daily time of measurement verified by written communication from an engineer on the official website of the National Oceanic and Atmospheric Administration (NOAA) at: ftp.cpc.ncep.noaa.gov/long/uv/cities/) was selected as a clinically relevant environmental metric, as UVI of 3 is the threshold of the Centers of Disease Control recommendation to apply sunblock [7].

SB locales within the US were defined as areas with latitudes <40 degrees based on the World Atlas (worldatlas.com/regions/sun-belt-states.html). NSB locales were defined as areas with latitudes >40 degrees. The inclusion of cities and years was based on the availability and completeness of UVI data from the publicly available NOAA database. Years from locales in which >10% of days were missing UVI data were excluded from analysis.

Available age-adjusted NMSC incidences were obtained from the Surveillance, Epidemiology, and End Results (SEER) Research Plus database (version 8.4.0.1, accessed November 30, 2022, for 18 registries) for the counties that the cities were located in. The most populous city with sufficient and available data within each county was selected as the fixed point from where UVI data was collected. Whenever possible, the counties with distinct latitudes were included in the study.

For this study, the NMSC incidences comprise only CSCC of the head and neck (CSCCHN) and MCC, as these are the only two types of NMSC in the SEER database. Nevertheless, CSCCHN is still clinically important since the head and neck locations comprise the majority of CSCC2. Data for age-adjusted CSCCHN and MCC incidences were available for the counties listed below from 2010 to 2019, but only 2010-2017 was included due to >10% missing UVI data for 2018 in at least one locale.

Five SB and four NSB locales from the SEER Research Plus database were identified with sufficient UVI, CSCC, and MCC data for analysis. SB locales included Bernalillo County, NM; Fulton County, GA; Honolulu County, HI; Los Angeles County, CA; and Orleans Parish, LA. NSB locales included Anchorage Municipality, AK; Atlantic County, NJ; King County, WA; and Salt Lake City, UT. Demographic information including age, gender, and race was collected from these locales.

The relationships between the percentage of days with UVI >3 each year and incidences of CSSCCHN, MCC, and combined CSCCHN and MCC were estimated by a linear mixed model adjusting, for the percentages of white race, male gender, age >55 years, and SB versus NSB locations, accounting for the repeated measures over the years using SAS, version 9.4 (SAS Institute Inc., Cary, NC, USA).

## Results

**Figure 1:**
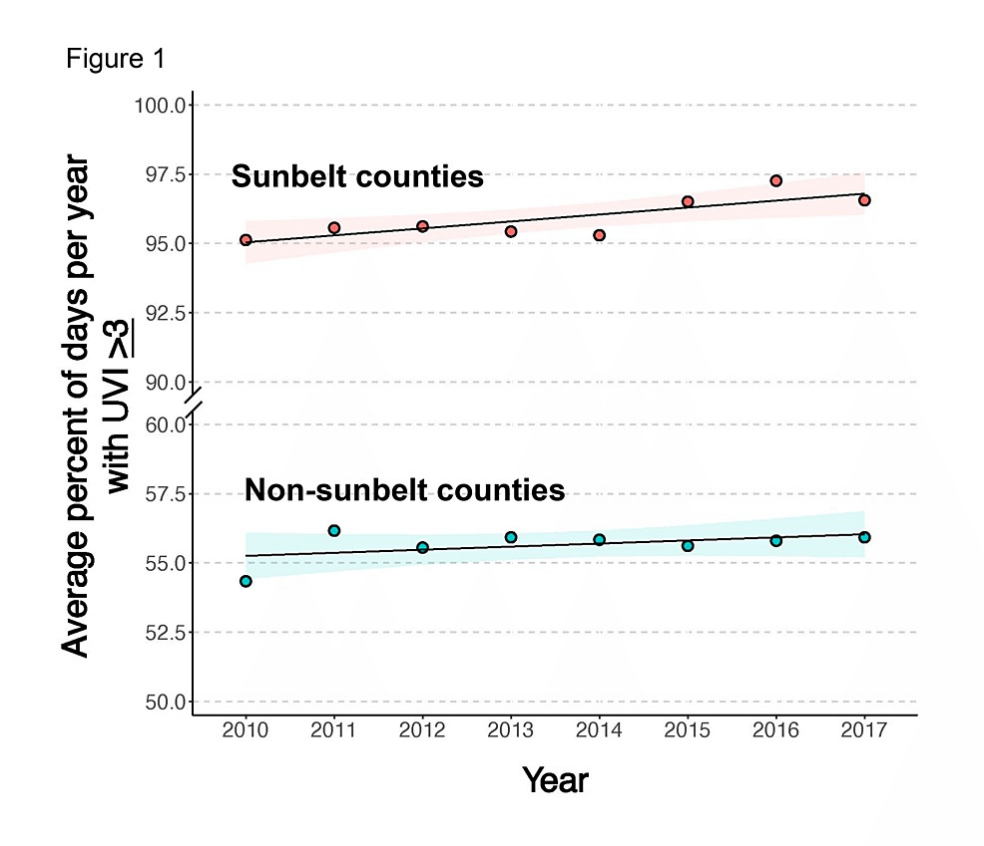
Average percent of days per year with ultraviolet index > or equal to 3 for the sunbelt and non-sunbelt counties included in this study from 2010 to 2017. There is an upward trend in the average percent of days with ultraviolet index >3 for both the sunbelt (n=5) and non-sunbelt (n=4) counties. Total counties included in study n=9.

The average percent of days per year with UVI >3 increased during the time period of this study, from 2010 to 2017, for both SB and NSB locales (Figure 1). 

**Figure 2:**
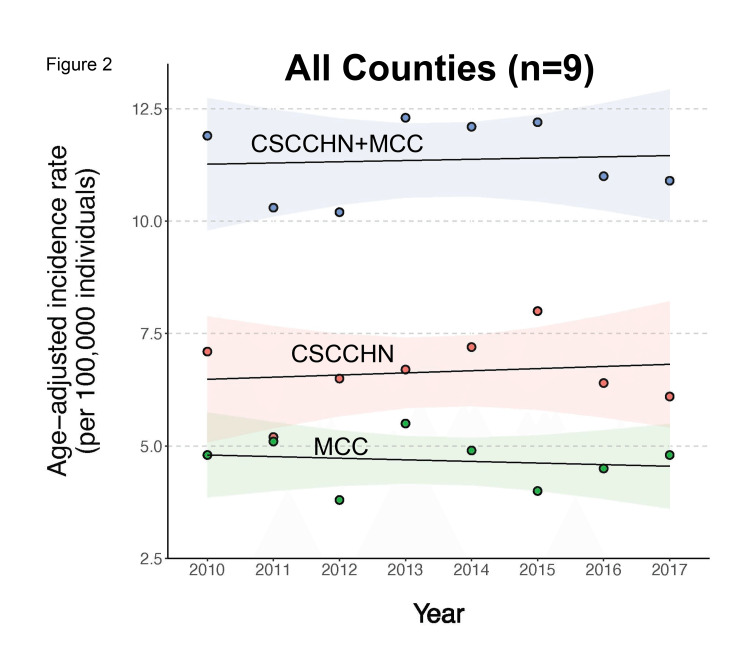
Combined cutaneous squamous cell carcinoma of the head and neck (CSCCHN) and Merkel cell carcinoma (MCC)) age-adjusted incidences (blue dots) increased slightly overall from 2010 to 2017 in the counties (n=9) in this study. Note that the increase in combined CSCCHN and MCC age-adjusted incidence (blue dots) over this period is driven by the individual CSCCHN age-adjusted incidence (red dots), since the individual MCC age-adjusted incidence decreased slightly over this period (green dots). Color-shaded areas indicate standard error.

Combined CSCCHN and MCC age-adjusted incidence across this same time period increased for all locales included in the study (Figure 2), though the individual MCC age-adjusted incidence did not increase. This is due to the predominant effect of the higher CSCCHN age-adjusted incidence rate compared to MCC age-adjusted incidence (MCC is a much rarer skin cancer type).

**Figure 3:**
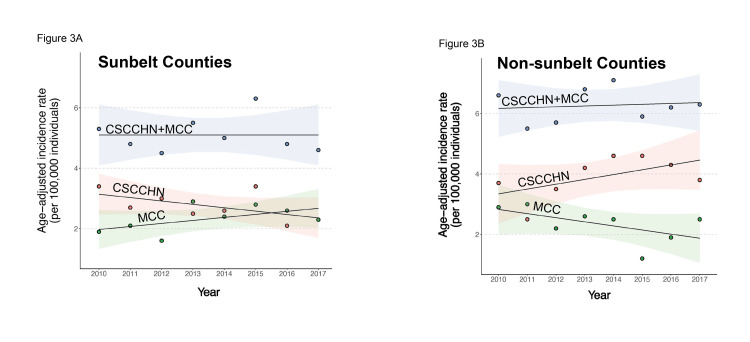
Sunbelt and non-sunbelt patterns of the age-adjusted incidence of cutaneous squamous cell carcinoma of the head and neck (CSCCHN) and Merkel cell carcinoma (MCC). (A) Combined CSCCHN and MCC age-adjusted incidence rate (blue dots) did not increase in sunbelt locales (n=5) from 2010 to 2017. Individually, the CSCCHN age-adjusted incidence decreased during this time, while the MCC age-adjusted incidence increased in sunbelt locales. (B) Combined cutaneous squamous cell carcinoma of the head and neck (CSCCHN) and Merkel cell carcinoma (MCC)) age-adjusted incidence rate (blue dots) increased in non-sunbelt locales (n=4) from 2010 to 2017. Individually, the CSCCHN age-adjusted incidence increased during this time, while the MCC age-adjusted incidence decreased in sunbelt locales. Color-shaded areas indicate standard error.

When separated by SB and NSB locales, the MCC age-adjusted incidence increased in the SB locales (Figure 3A), while the CSCCHN age-adjusted incidence increased in the NSB locales (Figure 3B).

**Figure 4:**
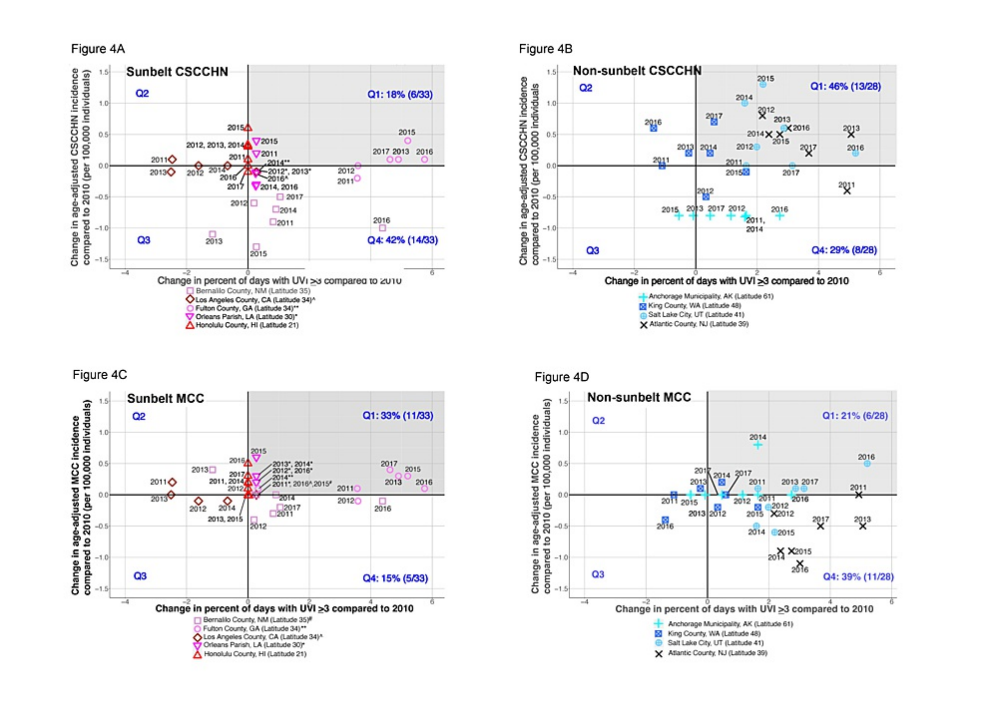
Change in the age-adjusted incidence of cutaneous squamous cell carcinoma of the head and neck (CSCCHN) or Merkel cell carcinoma (MCC) versus change in ultraviolet index (>/=3) percentage over time, compared to those in 2010 (the earliest year of this study) of sunbelt and non-sunbelt locales. (A) Changes in the age-adjusted CSCCHN incidence versus changes in the ultraviolet index (UVI) >/=3 percentage in the sunbelt counties over time compared to those in 2010. (B) Changes in the age-adjusted CSCCHN incidence versus changes in the UVI >/=3 percentage in non-sunbelt counties over time compared to 2010. (C) Changes in the age-adjusted MCC incidence versus changes in the UVI >/=3 percentage in sunbelt counties over time compared to those in 2010. (D) Changes in the age-adjusted MCC incidence versus changes in UVI >/=3 in non-sunbelt counties over time compared to those in 2010. Q=Quadrant

To assess these trends in more detail, the remaining data in this study will be presented to show individual counties. The patterns of changes in age-adjusted incidence rates versus changes in the percent of days with UVI >/=3 compared to 2010 data were plotted out for CSCCHN for individual SB counties (Figure 4A), CSCCHN for individual NSB counties (Figure 4B), MCC for individual SB counties (Figure 4C), and MCC for individual NSB counties (4D). 

**Figure 5:**
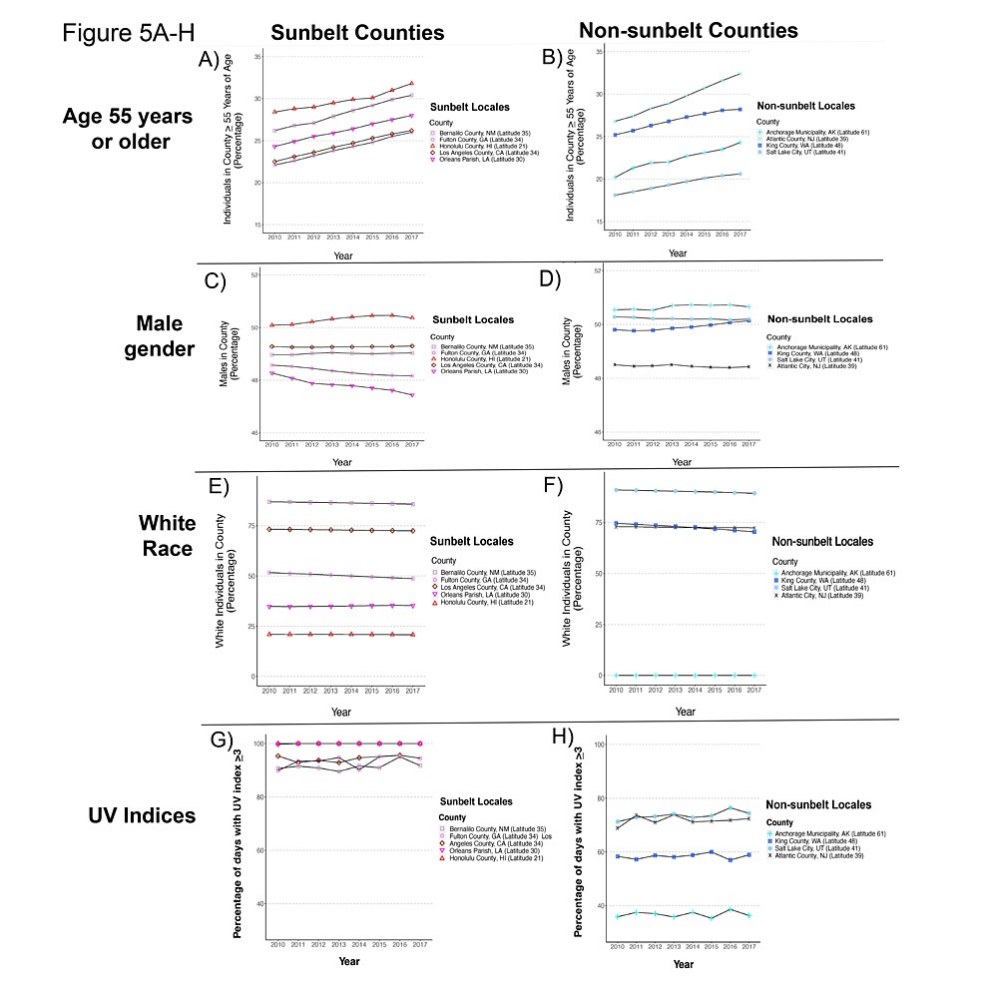
Demographic and ultraviolet characteristics of individual sunbelt and non-sunbelt counties. Demographic and ultraviolet characteristics of individual sunbelt and non-sunbelt counties. (A) Percentage of individuals aged 55 years or older in 2010-2017 in individual sunbelt counties and (B) non-sunbelt counties. (C) Gender composition in 2010-2017 of individual sunbelt counties and (D) non-sunbelt counties. (E) Racial composition in 2010-2017 of individual sunbelt counties and (F) non-sunbelt counties. (G) Percentage of days with ultraviolet index (UVI) >/=3 at high noon in 2010-2017 of individual sunbelt counties and (G) non-sunbelt counties.

NSB counties contain a higher percentage of data points (46% in Quadrant 1, or 13/28), in which both CSCCHN rates and percentage of days with UVI >/=3 increase over time (Figure 4B), than SB locales (18% in Quadrant 1, or 6/33) (Figure 4A). While NSB locales mostly increase their percentage of days with UVI >/=3 over the years compared to that in 2010 (Quadrants 1 and 4) (Figure 4B), the highest latitude county (Anchorage Municipality, indicated by teal crosses) showed a stable CSCCHN age-adjusted incidence over time, potentially due to the >75% non-white population in that county (see Figure 5).

The reasons for more data points in the SB counties demonstrating decreases in the CSCCHN age-adjusted incidence (42% (14/33) in Quadrant 4 (Figure 4A) over time, compared to 18% (6/33) in Quadrant 1 (Figure 4A) despite increases in percent UVI >/=3 over time, require further study and could be partially due to increasing percentages of non-white residents in the SB locales over time. However, other factors, such as increased sun safety behaviors among SB inhabitants in those counties, could also be at play.

A different pattern was observed for MCC age-adjusted incidences over time, in that SB counties demonstrated a higher percentage of data points in which both age-adjusted MCC incidence and percent of days with UVI >/=3 increased over time (Figure 4C, Quadrant 1, 33% (11/33)) compared to NSB locales (Figure 4D, Quadrant 1, 21% (6/28)), though, in subsequent statistical analysis, this was not significant (Table 1).

**Table 1:** Linear mixed model shows significant demographic and geographic factors impacting the incidence of cutaneous squamous cell carcinoma of the head and neck (CSCCHN) and Merkel cell carcinoma (MCC). Note that the percentage of individuals age 55 years or older is not significant because age-adjusted cancer incidences were used. Incidences are per 100,000 individuals. CI=confidence interval, UVI=ultraviolet index *For example, for each percent increase of the male gender, the CSCCHN rate increased to 0.320/100,000. **For example, for each percent increase of white race in the community, the CSCCHN rate increased to 0.009/100,000. ***For example, for each percent increase of UVI >3 at high noon per year, the CSCCHN rate increased to 0.021/100,000. ^For example, when combining CSCCHN and MCC rates, the incidence rate in the non-sunbelt locales is 1.227 (per 100,000) more compared to that of the sunbelt locales.

Covariate	CSCCHN		MCC		CSCCHN +MCC	
	beta coefficient (95% CI)	p-value	beta coefficient (95% CI)	p-value	beta coefficient (95% CI)	p-value
% persons aged >/= to 55 years	-0.017 (-0.056, 0.022)	0.3793	0.022 (-0.013, 0.056)	0.213	0.003 (-0.032, 0.038)	0.8535
% male	0.320* (0.170, 0.471)	<0.0001	-0.039 (-0.173, 0.095)	0.5585	0.287 (0.152, 0.421)	<0.0001
% white race	0.009** (0.004, 0.014)	0.0004	0.005 (0.001, 0.010)	0.0204	0.014 (0.010, 0.019)	<0.0001
% days with UVI >/= to 3 at high noon	0.021*** (0.006, 0.035)	0.0056	0.006 (-0.007, -0.019)	0.3536	0.028 (0.015, 0.041)	<0.0001
Non-sunbelt vs. sunbelt locale	0.781 (0.111, 1.450)	0.0283	0.399 (-0.194, 0.993)	0.1555	1.227^ (0.625, 1.829)	0.0019

Demographic and ultraviolet characteristics of individual SB and NSB counties are shown in Figure 5. Percentage of individuals aged >55 years increased for all SB and NSB counties over time (Figures 5A-5B) (note: we use age-adjusted CSCCHN and MCC incidences throughout this study). The percentage of males decreased in two SB counties and two NSB counties (Figures 5C-5D). The percentage of white individuals decreased in three SB and three NSB counties (Figures 5E-5F). The individual counties' UVIs >/=3 underpinning the increase in percentages per year over the study time period for both SB and NSB locales (previously shown in Figure 1) are displayed in Figures 5G-5H.

In linear mixed modeling, these demographic and ultraviolet characteristics, as well as NSB/SB location, were treated as covariates to determine their contribution to the CSCCHN, MCC, and combined CSCCHN and MCC incidences (per 100,000 individuals) (Table 1).

For each percent increase of UVI >/=3 at high noon per year, the age-adjusted CSCCHN incidence increased by 0.021 (per 100,000 individuals) (beta coefficient=0.021, p=0.0056) (Table 1). The NSB locales also significantly impacted the age-adjusted CSCCHN incidence, with a higher beta of 0.781 (p=0.0283). Percent male and percent white race also contributed to the age-adjusted CSCCHN incidence, with beta coefficient=0.320 (p<0.0001) and beta coefficient=0.009 (p=0.0004), respectively. The covariate of percent persons aged >/=55 years was not significant because age-adjusted incidences were used throughout the study.

Linear mixed modeling showed that only percent white race increased the age-adjusted MCC incidence (per 100,000 individuals), with a beta coefficient=0.005 (p=0.0204) (Table 1). 

Linear mixed modeling for the combined CSCCHN and MCC age-adjusted incidence paralleled the results of the CSCCHN age-adjusted incidence alone, with the same demographic factors contributing to the combined CSCCHN and MCC age-adjusted incidence as to the CSCCHN age-adjusted incidence. These factors were percent male (beta coefficient =0.287, p<0.0001) and percent white race (beta coefficient =0.014, p<0.0001). The geographic factors of percent of days with UVI >3 (beta coefficient =0.028, p<0.0001) and NSB locales (beta coefficient =1.227, p=0.0019) also contributed to the combined CSCCHN and MCC age-adjusted incidence (Table 1).

## Discussion

Our work is an initial step to integrate recent environmental data with demographic factors as contributing to NMSC incidences (in this study, specifically to CSCCHN and MCC incidences). Meshing of environmental and clinical factors is critical as ozone layers have decreased over the decades with concomitant increases in UVR reaching the earth’s surface [8]. Long-term health effects of these increased UVR doses would be manifest in the first organ system that the UVR encounters on the human body, the skin, as our data supports.

A few population-based reports in the medical literature exist that discuss the links between UV levels and skin cancer, but have focused on melanoma, and not NMSC. For instance, melanoma incidence has been associated with UV indices, though this data was not integrated with demographic factors [9]. In another study, melanoma, but not NMSC, has been studied with regard to UVI and latitude in non-white populations [10]. Studying geographic factors and NMSC incidences is all the more important since the CSCC incidence exceeds the melanoma incidence in the US by over ninefold [1, 11] and MCC is more aggressive than melanoma [12].

Separating locales into NSB and SB allowed us to discern differing trends for age-adjusted CSCCHN and MCC incidences. Our analysis in Table 1 showed that NSB versus SB locale was important for CSCCHN, but not for MCC age-adjusted incidences. The reasons for this difference require further study and are beyond the scope of our current study. For instance, there could be differing mechanisms underlying CSCC and MCC carcinogenesis, such as (1) chronic versus intermittent UV exposure with differential effects on white individuals, (2) the importance of polyomavirus (a non-geographic factor) in MCC carcinogenesis but not CSCCHN [13], or (3) differing doses of UVA or UVB to keratinocytes (which give rise to CSCCHN) versus Merkel cells based on their differing depths from the skin surface. In some countries from a variety of latitudes, age-adjusted MCC incidences have not increased over time, consistent with our data (see Denmark population data 1990-2007, or U. S. SEER data in non-white “other” population from 1990-2012) [14]. Our MCC data in Table 1 also demonstrated that the percent male gender reduced the age-adjusted incidence of MCC in our study locales, an effect reported in some populations, such as Ireland (1996-2007) and Finland (1994-2004) [14], though not consistent with the data from a larger number of locales in the US white population from an earlier time period (1990-2012) than our current study [14].

Our study is limited by the completeness and quality of data in the environmental and SEER databases. We may not have accounted for all covariates, such as lifetime cumulative UVR exposures, which are not feasible to assess in a retrospective manner in large populations. Furthermore, variation in UVI may exist within one county, especially if it is geographically large.

Despite the limitations, we believe that the trends in environmental or geographic factors and CSCCHN and MCC incidences described in our study are likely to continue and even increase in the future. Longer-term studies over more locales are needed to detect monitor and confirm our observations. Reversing these trends may require increased sun-safety education efforts targeted at specific locations, such as NSB locales, potentially under-recognized compared to SB locales as needing improved sun-safety education despite most of the US being in NSB locales [7], a topic requiring further study.

Furthermore, current national sun safety education efforts (examples shown in Table 2) may not consider a specific geographic locale or UVI patterns. Integrating this environmental information (as well as demographic factors) could lead to more effective prevention campaigns, since recommendations for the population in one locale (e.g. SB) may not necessarily be perceived as relevant for the population in another locale (e.g. NSB). Furthermore, within the US, there may be differing levels of sun-safety knowledge, attitudes, and practices depending on the locale, based on historical data (pre-2010) that may no longer be up-to-date.

**Table 2:** Examples of current evidence-based sun-safety programs for adults from the National Institutes of Health, but are not necessarily locale-based. The text in the column labeled “Purpose” of studies was taken from the Evidence-Based Cancer Control Programs website, accessed at ebccp.cancercontrol.cancer.gov/topicPrograms.do?topicId=102269&choice=default.

Program Title and Description	Target Age (years)	Purpose	Program URL
Appearance-focused Skin Cancer Prevention Intervention	19-39	To reduce indoor tanning through the awareness of the harmful effects of exposure to UV radiation.	https://ebccp.cancercontrol.cancer.gov/programDetails.do?programId=310543
Go Sun Smart (GSS)	19+	To promote sun-safety practices to ski area employees.	https://ebccp.cancercontrol.cancer.gov/programDetails.do?programId=308006
Project SUNWISE: Skin Cancer Prevention Counseling by Pharmacists	19-65	To increase the effectiveness of pharmacists in delivering skin cancer prevention counseling.	https://ebccp.cancercontrol.cancer.gov/programDetails.do?programId=282372
Sun Protection Strategies for Kidney Transplant Recipients	19+	To increase awareness and promote sun protection behavior and practices among kidney transplant recipients.	https://ebccp.cancercontrol.cancer.gov/programDetails.do?programId=26162780
Sun Safety Among U.S. Postal Service Letter Carriers ("Project SUNWISE")	19-65	To promote sun-safety practices to postal service letter carrier employees.	https://ebccp.cancercontrol.cancer.gov/programDetails.do?programId=313055
Together for Sun Safety	19-65	To motivate sun protection behavior and reduce sun exposure.	https://ebccp.cancercontrol.cancer.gov/programDetails.do?programId=229166

## Conclusions

When age-adjusted CSCCHN and MCC rates are combined from 2010 to 2017, the overall rate is significantly correlated with NSB locales. This is likely driven by age-adjusted CSCCHN rates, since age-adjusted MCC rates alone do not show this effect. Hence, the NSB locales should not be overlooked in sun-safety outreach efforts, especially in light of data that ultraviolet indices at levels where sunblock is recommended (ultraviolet index greater than or equal to 3) at high noon are increasing in frequency in both NSB and SB locations. Future research is needed to determine whether dermatologists should increase sun-protective behavioral counseling for patients in NSB locations, or whether patients in NSB locations under-estimate the importance of sun-protective behaviors.
